# Exponential feedback effects in a parametric resonance climate model

**DOI:** 10.1038/s41598-023-50350-7

**Published:** 2023-12-27

**Authors:** Maria Teresa Caccamo, Salvatore Magazù

**Affiliations:** 1 Dipartimento di Scienze Matematiche e Informatiche, Scienze Fisiche e Scienze della Terra, Università di Messina, Viale Ferdinando Stagno D’Alcontres n°31, S. Agata, 98166 Messina, Italy; 2 Consorzio Interuniversitario Scienze Fisiche Applicate (CISFA), Viale Ferdinando Stagno D’Alcontres n°31, S. Agata, 98166 Messina, Italy

**Keywords:** Climate change, Mathematics and computing, Computational science

## Abstract

The variations in the distribution of solar radiation due to the  ~ 10^5^ years Milankovitch cycle, which is connected to the Earth eccentricity variation, cannot explain the sharp drop in temperature of 6 °C ÷ 10 °C that marks the transition from the interglacial to the glacial age registered in the last ~ 5.5 10^6^ years temperature variation behavior. More specifically, neglecting other effects, only a temperature variation of 0.2 °C ÷ 0.3 °C can be attributed to this cycle and, therefore, positive feedback effects should be taken into account to explain the registered effect. In the present work, a comparative Wavelet-Fourier analysis of the Vostok recontructed temperature record, for which different sampling steps are taken into account, is performed. Then, a study of exponential feedback effects within a climate parametric resonance model is dealt and discussed. The obtained findings put into evidence an exponential amplification of the temperature variation from the interglacial to the glacial age supporting the hypothesis that the system energization be connected to periodic variations in the internal solar system parameters. More in details, it is shown that, following the parametric resonance climate model, even small oscillations increase over time proportionally to the system energy itself, i.e. exponentially, and hence, a series of connected resonances is able to energize the climate system.

## Introduction

Since ancient times, people understood the connection between solar energy flow and daily and seasonal temperature variations as evidenced by the Greek word "klima", which originally referred to the inclination of the Sun's rays with respect to the Earth's tangent plane at a given latitude.

Starting from the end of the nineteenth century, breakthroughs in physical sciences have made it possible to analyse the basic mechanisms underlying air and ocean motions and to evaluate the Earth’s radiative balance. The latter was shown to be mainly governed by the flow of energy between the Earth and space which is dependent on both the temperature of the Earth's surface and on the greenhouse effect, originally described in 1824 by the French scientist Joseph Fourier.

In particular, the greenhouse effect recalled the attention on the contribution of human-emitted CO_2_ quantity and inspired the Swedish scientist Svante Arrhenius to make a prediction about the evolution of our planet's temperature, later confirmed by geologists, glaciologists, and other natural scientists^1–3^.

On the other hand, the measurement of the abundance of oxygen isotopes, preserved in marine sediments and in glacier ice, provided a precise testimony of the Earth temperature variations during the Pleistocene^4–11^. In particular, pleistocene marine sediments contain numerous fossils of foraminifera that are small single-celled marine organisms that secrete shells of calcite (CaCO_3_). The amount with which the different oxygen isotopes are incorporated into these shells depends on the isotope ratio of the seawater in which these organisms lived. By measuring the ^18^O/^16^O isotope ratio in different layers of marine sediments, paleoclimatologists^12–14^ have obtained information on surface water temperatures and the volume of ice at the time of sediment deposition. Therefore, it is possible to extract the planet's temperature variations by monitoring the ice ^18^O/^16^O ratio^14,15^.

Dansgaard et al.^16^ have investigated the correlations between the temperature of air and the temperature of the phase transition precipitations putting into evidence that uncertainties arise in reconstructing temperature data from the isotopic composition of Antarctic ice stations. More specifically, while precipitations have their origin mainly from the Atlantic, Pacific and Indian Oceans, the isotopic composition of the air masses can remarkably vary during air displacements from one region to another; on the other hand, storm transfer of falling snow and firnization effects, with freezing and thawing phases, are often accompanied by considerable mass transfer that can significantly distort the isotopic composition distribution. Furthermore, ice diagenesis can induce significant transformation of the isotopic composition of ice while, under pressure, the formed subglacial rivers and lakes may give rise to huge mass exchanges. As a result, ice core analyses can furnish values that, due to these uncertainties, are not clearly attributable.

**Figure 1:**
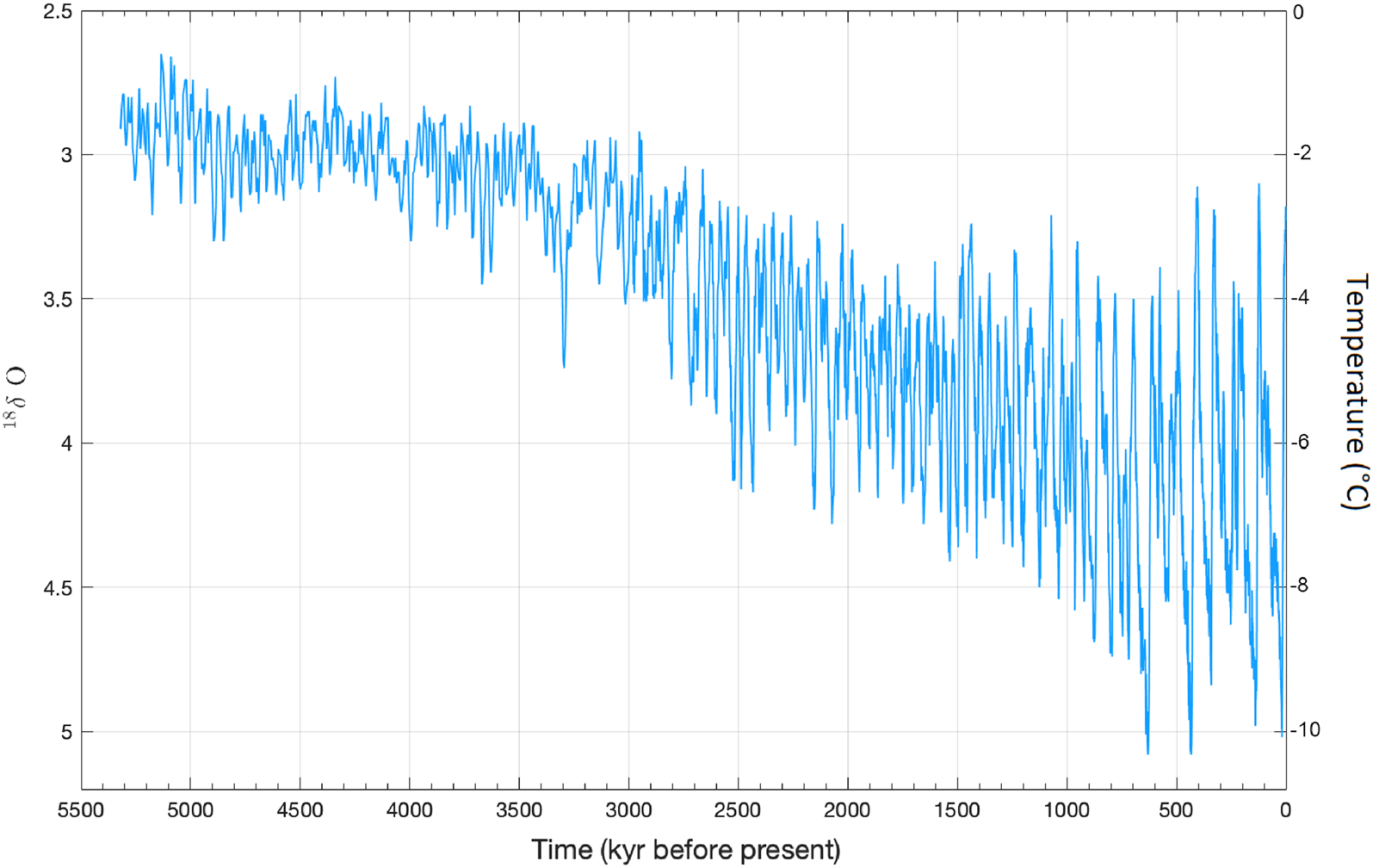
Time behavior of the ^18^δO concentration^14^ and of the Earth’s temperature during the last 5.5 Myr BP.

Figure 1 reports the behaviour of the O^18^ concentration as a function of time over the last 5.5 Myr Before Present (BP)^14,15^. It emerges that in the Pleistocene, spanning the time period from 11,700 years to 2.58 Million years ago (Myr), some of the most striking climate change signatures are observed. More specifically, periodic variations in O^18^ concentration as a function of time are registered that have been interpreted as a sign of the alternation of glacial and interglacial climate cycles. In particular, glacial cycles result asymmetric in time, with slow cooling from interglacial to glacial states and quick warming from glacial to interglacial states; this fast-slow dynamics is referred to as the "saw-tooth" form of the paleoclimatic record. In particular, beginning with a slow drop in temperature, a glacial cycle results in an increase in sea ice, polar ice cap area and volume and a decrease in ocean water volume with emerging continental portions.

These processes increase the amount of radiation reflected out by the Earth (i.e., albedo) and thus cause a further drop in temperature (positive feedback). A rise in temperature signals the end of a glacial age, which in turn results in an increase in the available water from the cryosphere^15,17^.

Concerning the alternation of glacial-interglacial periods, it should be considered that, due to the gravitational interactions between the Earth and the other bodies of the solar system, the characteristics of the Earth's orbit (i.e. the Earth astronomical parameters) are not fixed, but show regular variations. Three main astronomical parameters can be identified: the eccentricity of the Earth's orbit, the obliquity of the axis of rotation and the precession. Precursors such as Adhémar and Croll in the nineteenth century, and then Milankovitch in the 1920s to 1940s, suggested a link between these astronomical parameters and the alternation of glacial and interglacial periods that is known as the astronomical theory of paleoclimates which still constitutes the current paradigm.

In particular, Milankovitch suggested that the Earth's climate is influenced by three different cycles: the periodic eccentricity variation of the Earth's orbit, with a period of  ~ 10^5^ years; the Earth's axis oscillation, with a period of  ~ 45 Kyr; and the cyclic wobbling of the Earth's axis, with a period of ~ 23 Kyr.

It should be stressed that temperature changes are mainly associated with variations in incoming summer radiation which are determined not only by changes in the Earth eccentricity orbit, but also by variations in the inclination of the axis; as far as the Stefan-Boltzmann law is concerned, the extreme values of summer insolation correspond to a temperature variation of about 3.85 °C, and, taking into account the unequal albedo for the extreme conditions, this temperature variation can increase. Oceans influence climate in various ways. In particular, ocean currents transport a significant amount of heat, usually directed poleward and thus contributing to a reduction of the pole to equator temperature gradient. However, these effects have short characteristic times, and therefore their influence should be negligible.

**Figure 2:**
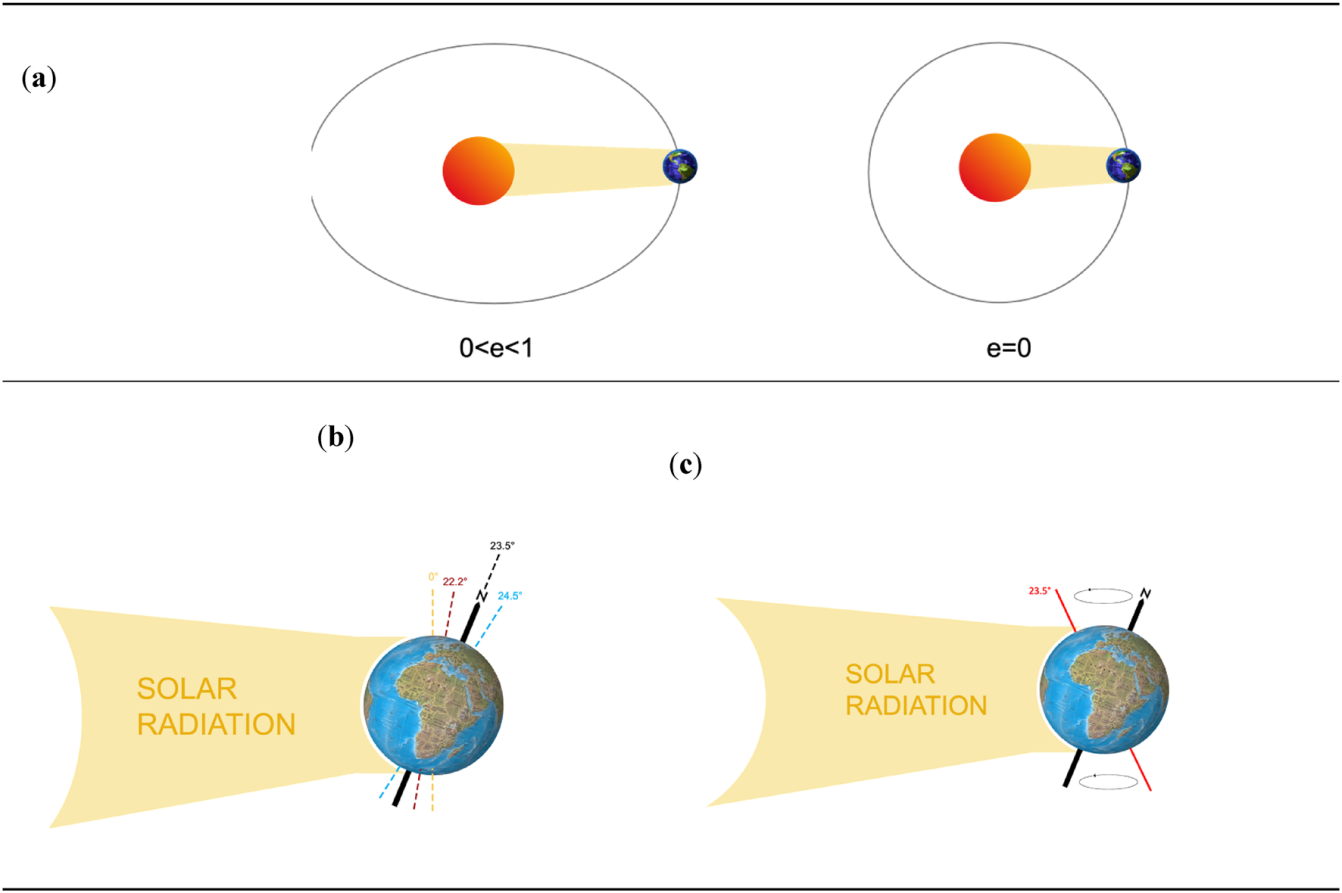
Milankovitch cycles: (**a**) Cycle connected with the Earth’s orbit eccentricity variation with a period of ~ 100 Kyr; (**b**) Cycle connected with the Earth’s axis inclination variation, from 21° 55′ to 24° 20′, with a period of ~ 41 Kyr; (**c**) Cycle connected with the Earth’s axis precession, i.e., with the double-cone Earth’s axis motion, with a period of ~ 23 Kyr.

A sketch of the three Milankovitch cycles is reported in Fig. 2. In particular, Fig. 2a shows the Earth's eccentricity variation, which has a period of approximately 100 Kyr, the Earth's orbital eccentricity ranging from  ~ 0 to  ~ 0.07, with a current value of  ~ 0.017.

The solar constant is 1361 W/m^2^ but, due to the eccentricity of the orbit, the flux received by the Earth at normal incidence currently varies by 6% during the year, between 1408 W/m^2^ at perihelion and 1317 W/m^2^ at aphelion. The variations in eccentricity over geological time may have led to sometimes much greater variations (the current value of eccentricity is 0.0167, or 3.5 times less than the maximum value of eccentricity of 0.06). This parameter varies with a periodicity close to 100,000 years (95 and 125 Kyr to be exact). There is also a variation of the eccentricity close to 400,000 years (413 Kyr precisely). Furthermore, the average distance between the Sun and the Earth is ~ 1.49 10^11^ m, whereas the Sun's average diameter is ~ 110 times that of the Earth (12.750 10^3^ m). One of the key issues of the astronomical theory of ice age cycles is the so called 100 kyr problem^18–24^. While the Northern Hemisphere summer insolation, which is assumed to be the driver of ice age cycles in the Milankovitch theory, has only a negligible spectral power contribution at ~ 100 Kyr, the climate system oscillates more strongly at  ~ 100 Kyr^25,26^.

Figure 2b depicts the cycle of the Earth axis inclination variation, which has a period of  ~ 41 Kyr within a range of 21° 55′ to 24° 20′. The seasons are caused by the tilt of the Earth's axis with respect to the orbital plane. The amount of solar radiation that hits different portions of the Earth varies with this tilt variation. Today, the Earth's axis is inclined at ~ 23.5°, and this value becomes less skewed over time.

Finally, the movement in the Earth's axis is caused by a double cone motion, that is a precession motion, with a period of ~ 23 Kyr (see Fig. 2c). The orientation of the Earth's axis is altered by this precession motion rather than its inclination. For millennia, the Earth's axis has pointed toward Polaris, often known as the North Star. However, the Earth was more in line with Thubin approximately 5 Kyr ago, and the axis of the Earth will line up with Vega in approximately 12 Kyr. The orientation of the planet with respect to aphelion and perihelion shifts after one cycle of precession: if one hemisphere faces the Sun during perihelion, it will be inverted during aphelion, and the same is true for the other hemisphere. The hemisphere that faces the Sun at perihelion and away from it at aphelion would therefore experience higher seasonal contrasts than the opposite hemisphere. The southern hemisphere currently experiences winter near aphelion and summer near perihelion, meaning that it has more extreme seasons than the northern hemisphere^27–29^.

It should be taken into account that Milankovitch’s cycle periods vary significantly over time. Fedorov et al. have evidenced that the climatic precession cycle, according to the ephemerides of Laskar et al.^30^calculated for 5 million years in the past and 5 million years in the future varies from 10,000 to 33,000 years. A similar situation is observed for the axis tilt cycle, where the period varies from 33 to 52 thousand years. Finally, an analogous behaviour is present for eccentricity whose cycle in the interval from 5 million years in the past to 5 million years in the future will decrease by 30%. Taking these indicators into account can help to obtain more complete information about climate changes in past eras and can allow more accurate future climate forecasts^31–33^. As a result of these variations, spectral analysis of reconstructed temperature data can furnish significant distorted results relative to reality.

Knowing how Earth's environment has converted the heat input from the Sun into Earth temperature changes can help anticipating future global climate change, making it important to understand the climate of the recent geological past.

It should be also mentioned that temperature variations are also associated with changes in the intensity of radiative heat transfer; for example, global climatic events in the Holocene and late Pleistocene were synchronized with extreme values of variations in incoming summer solar radiation and variations in the intensity of radiative heat transfer^34–36^.

However, the climatic reaction to insolation fluctuations is far from trivial and feedback based models are to be taken into account. In particular, it is unclear how the climate system works and how modest variations in solar radiation at the top of the atmosphere may be amplified by the Earth system to produce the remarkable climatic shifts associated with glacial-interglacial cycles. Ice age cycles require a rearrangement of the ocean-atmospheric system, of the deep ocean and of its sedimentary interface, of the ocean chemistry, of the carbon cycle, of the terrestrial and marine ecosystems, and so on. The entire Earth is involved in the dynamics of ice ages in a complex fashion, and its components are linked together by a network of feedbacks.

Understanding the mechanisms underlying the ice ages and the most recent alterations in climate systems is becoming a crucial scientific topic in the contemporary context of anthropogenic global warming. While some attempts extend back decades, serious attempts to develop quantitative climate theories may be regarded to have started in the late 1960s.

Many numerical models have tried to reproduce the profile of glacial–interglacial cycles, most of them including a huge number of tunable parameters. However these theories of global climate cannot be translated into accurate numerical projections of climate decades in the future^37^. Nonetheless, a lot of issues may be reframed into understandable conceptual frameworks.

The goal of this paper is to show how within a simplified climate phenomenological model, based on a parametric resonance effect, an exponential amplification takes place which can furnish a comprehensive picture of the global climate system.

As shown by the impressive work of Benzi et al.^38^, the abrupt reduction in temperature that characterizes the change from an interglacial to a glacial age cannot be solely attributed to differences in the distribution of solar energy throughout the  ~ 10^5^ years Milankovitch cycle; therefore, it is plausible that positive feedback effects must be taken into account to explain the increase in the impact of this cause.

In this framework, Benzi et al.^38^ proposed a stochastic resonance effect as a solution to this conundrum. In contrast to linear systems, where the output is maximized in the absence of noise, this effect affects nonlinear systems that show an energy threshold (i.e., activation barrier) and is characterized by the fact that the system's output (i.e., the system response) to a weak input signal undergoes resonance-like behavior, i.e. to an output maximum for a given noise level.

The complex global climate system arises from the interaction of five systems, each of them being also a complex system, interacting together. These are: (i) the atmosphere, i.e. the layer of gases surrounding the Earth; (ii) the lithosphere, i.e. the land surfaces, such as soil and rocks, and human-made surfaces such as roads and buildings; (iii) the hydrosphere, i.e. the Earth’s liquid water in oceans, rivers, lakes and underground; (iv) the cryosphere, i.e. the frozen water in ice and snow; (v) the biosphere, i.e. the living beings such as plants and animals including humans.

Because the last decades have registered the hottest temperature variation in instrumentally recorded data history with a registered temperature rise particularly significant in the so-called hot spot or sentinel regions, in recent years, we have analysed the climate dynamics connected with the global Earth temperature increase. More specifically, we have investigated the stochastic resonance scenario under the assumption of an increase in temperature. Here, the outcomes of the conducted numerical simulations showed that a temperature increase results in a change from a stable to a chaotic regime.

In the present work, we discuss a parametric resonance model for climate, according to which the system energization is due to a periodic variation of Earth orbital parameters. In has been shown how, following this climate model, the energy variation in the system, although small, increases over time proportionally to the system energy and therefore exponentially^39,40^.

## Parametric resonance model

This section provides a straightforward description of the parametric resonance model through a simple device consisting of a pendulum whose length is variable according to a sinusoidal law with variable frequency. In particular, it will be shown that the energy in the chosen model system increases over time exponentially, namely with a speed proportional to the energy of the system itself.

It is well known how, according to a conventional classification, the oscillations are free when the oscillating system is left to itself; in this case, in the presence of friction, due to the dissipation of energy, the oscillations gradually decay over time.

On the other hand, oscillations are classified as forced when the oscillator is subject to an external periodic force. In this case, after a transient phase, the forced oscillations become stationary and acquire the same frequency as the external force. Furthermore, when the frequency of the external force is close to the natural frequency of the oscillator, the amplitude of the oscillations can reach significant values giving rise to a phenomenon of resonance. Another way to energize the system oscillation can be obtained through a suitable periodic variation of the system parameters. This phenomenon is called parametric resonance since in this case, the origin of the resonance is due to a variation in the internal system parameters. A common example of a parametric resonance phenomenon is provided by a child moving on a swing. In this case, the effect is due to the variation in the effective length of the pendulum when this variation is performed with the right phase, for example, when the child squats his body at the extreme points of his oscillatory movement (generating an increase in the effective length of the pendulum) and then stretches out passing from the central position (generating a decrease in the effective length of the pendulum).

Therefore, the causes of parametric resonance are different from those of simple resonance, where the oscillator responds to a periodic external stress. Furthermore, parametric resonance only occurs if there is already a weak oscillation in the system and when the amplitude of the parameter exceeds a certain threshold value.

The parametric resonance treated in this section takes as a model system a pendulum when its length changes periodically and with variable frequencies. It is well known that the differential equation describing the movement of a simple pendulum of mass ${\varvec{m}}$:

1 $$m\mathcal{l}(t)\frac{{d}^{2}\theta }{d{t}^{2}}=-mgsen\theta (t)$$

**Figure 3:**
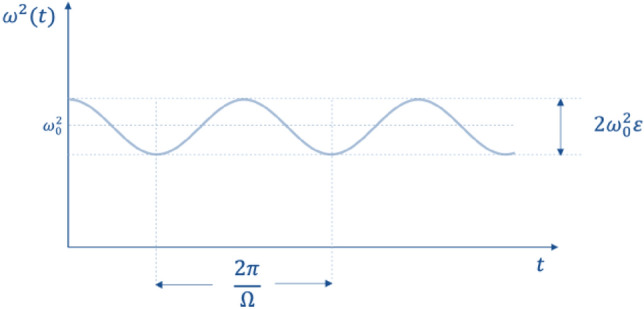
Time periodic variation of the *ω*^2^(*t*) parameter*.*

If the rate of change of the length is not negligible in respect to the rate of change of the angle, $\dot{\mathcal{l}}\left({\varvec{t}}\right)\sim \dot{{\varvec{\theta}}}\left({\varvec{t}}\right)$, then it must be taken into account that the length of the pendulum $\mathcal{l}({\varvec{t}})$ cannot be treated as a constant; therefore, it is necessary to consider the differential equation with variable coefficients. In such a case the equation can be put under the form:

2 $$ \frac{{d^{2} \alpha }}{{dt^{2} }} + \omega^{2} \left( {\text{t}} \right)\alpha = 0 $$

where *ω(t)* is the pulsation coefficient. With periodic variations in the parameter ${\varvec{\omega}}({\varvec{t}})$*,* the corresponding differential equation is designated Hill's equation. When the amplitude of the oscillation, caused by the periodic modulation of the internal parameter, increases, we refer to the phenomenon as parametric resonance. Let us suppose that the function describing the time variation of ${\varvec{\omega}}({\varvec{t}})$ is ${{\varvec{\omega}}}^{2}\left({\varvec{t}}\right)={{{\varvec{\omega}}}_{0}}^{2}(1+{\varvec{\epsilon}}{\varvec{c}}{\varvec{o}}{\varvec{s}}{\varvec{\Omega}}{\varvec{t}})$ con ${\varvec{\epsilon}}\ll 1$, i.e., Its variation is periodic and relatively small (see Fig. 3).

The equation of motion then becomes:

3 $$ \frac{{d^{2} \alpha }}{{dt^{2} }} + \omega_{0}^{2} \left( {1 + \epsilon \cos \Omega t} \right)\alpha = 0 $$

which is a particular type of differential equation with nonconstant coefficients called the Mathiew equation. This physical system is not conservative, as its total energy changes over time. As mentioned above, in particular, under certain conditions, the energy of the system will progressively increase while the oscillatory motion is increasingly amplified, giving rise to the phenomenon of parametric resonance. Parametric resonance can be shown to occur when the frequency of the *Ω* parameter is close to the following values:${\boldsymbol{\Omega }}_{{\varvec{n}}}=\frac{2{{\varvec{\omega}}}_{0}}{{\varvec{n}}}$ (n = 1,2,3,…) and that the maximum effect occurs when it is ${\varvec{n}}=1$, i.e., ${{\varvec{\Omega}}}_{\mathbf{n}}=2{{\varvec{\upomega}}}_{0}.$ In other words, the most significant parametric oscillations occur when the modulation cycle is repeated twice during a period of natural oscillations; however, resonance is also possible when the parameter changes once during a period, twice during three periods, and so on.

An important distinction between resonance induced by an external stress and parametric resonance is that for forced excitations, the increase in energy during a period is proportional to the amplitude of the oscillation, i.e., to the square root of the energy, for parametric resonance, the increase in energy is proportional to the energy stored in the system.

**Figure 4:**
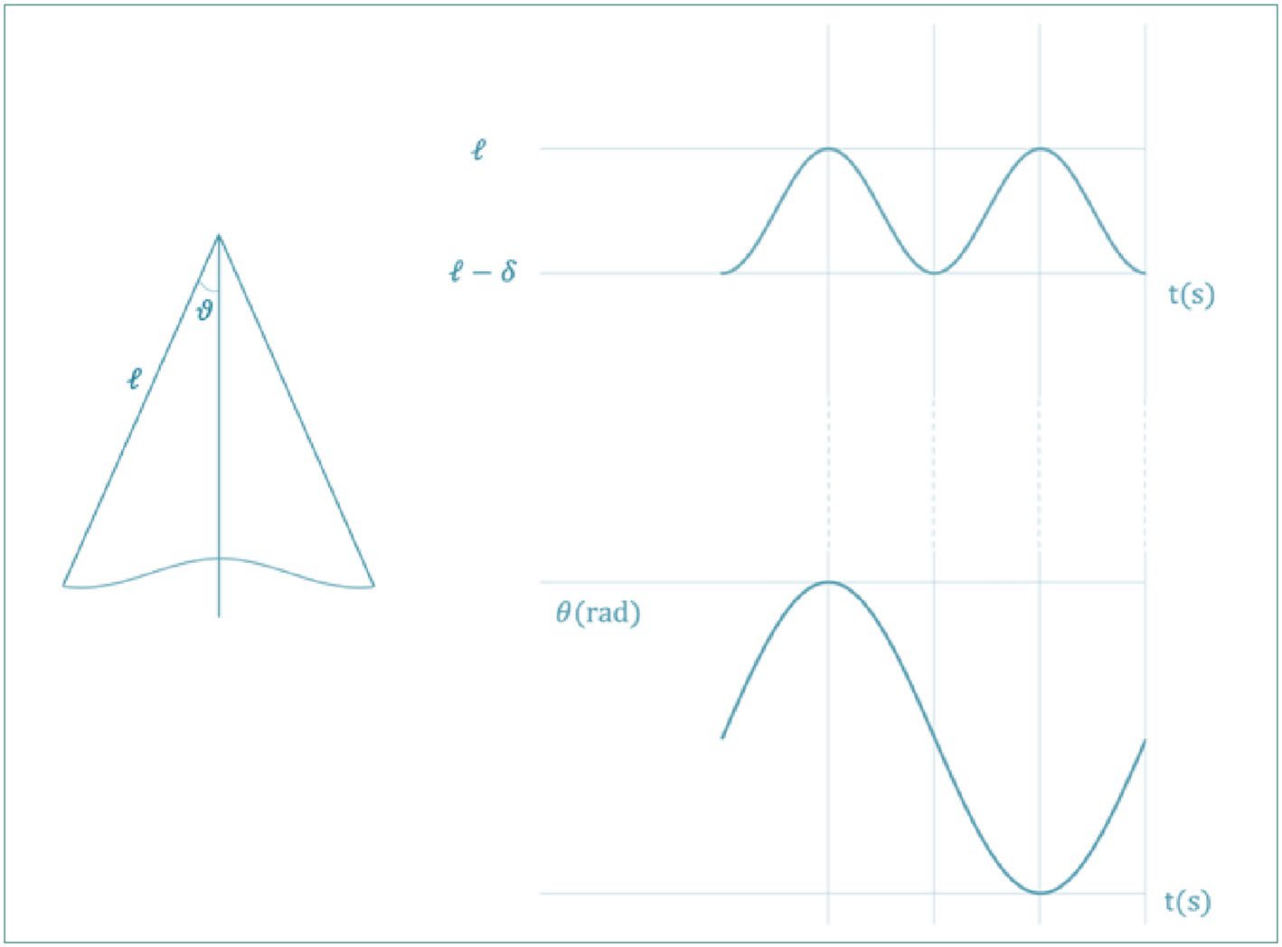
On the left: sketch of the variable length pendulum movement during the half period; top right: length as a function of time, $\mathcal{l}(t)$*;* bottom right: oscillation angle $\theta (t)$ versus time.

As shown in Fig. 4, to detect the phenomenon when ${\varvec{n}}=1$, it is necessary to vary the length of the pendulum periodically and with a frequency double the natural one. From the figure, the following can be seen:

(i) When the pendulum passes from the equilibrium position, there is a minimum length equal to ${\mathcal{l}}_{{\varvec{O}}}=\mathcal{l}-{\varvec{\delta}}$.
(ii) When the pendulum passes from the positions of maximum angular excursion, there is a maximum length equal to l.

Figure 4 shows a sketch of the movement of the variable length pendulum during the half period (on the left), of the change in length $\mathcal{l}({\varvec{t}})$ as a function of time (top right) and of the change in the oscillation angle ${\varvec{\theta}}({\varvec{t}})$ versus time (bottom right).

Indicating with ${{\varvec{v}}}_{{\varvec{O}}}$ and ${{\varvec{\theta}}}_{{\varvec{m}}}$ the maximum values of velocity and of the oscillation angle of the pendulum, one has:

i)i)i) When the length decreases, with a maximum decrease corresponding to the vertical position, the gravitational potential energy varies by:

4 $$ \Delta U_{1} = mg\delta $$

and, indicating with ${{\varvec{v}}}_{{\varvec{O}}}$ the maximum speed value in correspondence to the vertical position, its potential connected to the centrifugal force varies by:

5 $$\Delta {U}_{2}=\frac{m{v}_{0}^{2}}{\mathcal{l}}\delta $$

These variations correspond to work performed against the force of gravity *mg* and against the centrifugal force, which in the vertical position is equal to $\frac{{\varvec{m}}{{\varvec{v}}}_{0}^{2}}{\mathcal{l}}.$ When the length increases, with a maximum increase in the positions of maximum angular excursion, the gravitational potential energy variation.

for small angles, being $\mathbf{cos}{\varvec{\theta}}\cong 1-\frac{{{\varvec{\theta}}}^{2}}{2}$, can be expressed as:

6 $$\Delta {U}_{3}=-mg\delta \left(1- \frac{{\theta }_{m}^{2}}{2}\right)$$

Therefore, for every half cycle, there will be a net change in energy $\boldsymbol{\Delta }{\varvec{U}}$:

7 $$\Delta U=\Delta {U}_{1}+\Delta {U}_{2}+\Delta {U}_{3}=mg\delta \frac{{\theta }_{m}^{2}}{2}+\frac{m{v}_{0}^{2}}{\mathcal{l}}\delta $$

which is proportional to ${\varvec{\delta}}.$

As far as the system energy is concerned ${\varvec{E}}$, it is:

8 $$ \begin{aligned} & E = \frac{1}{2}mv_{0}^{2} = mg\ell \left( {1 - cos\theta_{m} } \right) = \\ & mg\ell \left[ {1 - \left( {1 - \frac{{\theta_{m}^{2} }}{2}{ }} \right)} \right] = mg\ell \frac{{\theta_{m}^{2} }}{2} \\ \end{aligned} $$

Therefore:

9 $${v}_{0}=\sqrt{g\mathcal{l}{\theta }_{m}^{2}}=\sqrt{\frac{\mathcal{l}}{g}}\mathcal{l}{\theta }_{m}$$

In conclusion, one has:

10 $$\Delta U=mg\delta \frac{{\theta }_{m}^{2}}{2}+\frac{m{v}_{0}^{2}}{\mathcal{l}}\delta =3\frac{\delta }{\mathcal{l}}\frac{1}{2}m{v}_{0}^{2}=3\frac{\delta }{\mathcal{l}}E$$

which is proportional to the energy of the system.

Therefore, the energy in the system increases over time with a speed proportional to the energy of the system itself and therefore exponentially. As a result, the amplitude of oscillation will increase over time.

Note that if the external perturbation acts with an opposite phase, that is, if the length increases when the pendulum moves towards the vertical position and decreases when it moves towards the position of maximum angular excursion, the energy of the system decreases, and the oscillations are dampened.

Finally, when the system’s friction force is not negligible, the solution of Eq. (6) prescribes a decrease of the frequency ranges within which parametric resonance effects occur. More specifically, concerning the double frequency parametric resonance condition, the resonance frequency window extends between ${2\omega }_{0}-\frac{1}{2}\epsilon {\omega }_{0}<$ ω $<{2\omega }_{0}+\frac{1}{2}\epsilon {\omega }_{0}$; in the presence of friction the solutions include multiplicative exponential damping factors ${e}^{{\alpha }_{i}t}$ and these terms give rise to a decrease of the resonance windows.

## Climate parametric resonance model

To model the climate behavior that gives rise to the behavior trend registered by the Vostok site, we start from the stochastic differential Langevin’s equation, which takes into account a systematic contribution, given by a time-dependent reactive force ${f}_{reactive}$, by a dissipative friction term ${f}_{dissipative}$, and by a stochastic term ${f}_{random}$^38–44^:

11 $$m\frac{{d}^{2}x(t)}{d{t}^{2}} ={f}_{reactive}+{f}_{dissipative}+{f}_{random.}$$

In other words, the climate system is modelled as a particle of mass m, with a given configuration coordinate x(t). Such a mathematical model can allow us to describe the temperature behaviour when one identifies the system configuration coordinate $x\left(t\right)$ with the Earth’s temperature $T\left(t\right).$

In particular, the climate system experiences^45–49^:

1. A time-dependent reactive force:

12 $${{f}_{reactive}\left(t\right)=-\upomega }^{2}\left(t\right)x={-\upomega }_{0}^{2}\left[1+\varepsilon cos\left({\Omega }_{0}t+\varphi \right)\right]x={-\upomega }_{0}^{2}x-{\upomega }_{0}^{2}\varepsilon cos\left({\Omega }_{0}t+\varphi \right)x$$

this term acts as an “energy” source and will parametrically excite the system.

2. A friction or Stokes’ force:

13 $${f}_{dissipative}=-\beta \frac{dx\left(t\right)}{dt}$$

3. And a random force connected with the system “heat bath”:

14 $${f}_{random}=\xi (t)$$

So, Eq. (11) becomes:

15 $$m\frac{{d}^{2}x(t)}{d{t}^{2}} ={-\upomega }_{0}^{2}\left[1+\varepsilon cos\left({\Omega }_{0}t+\varphi \right)\right]x-\beta \frac{dx\left(t\right)}{dt}+\xi \left(t\right).$$

In the following, we assume that the random force is negligible, i.e., $\xi \left(t\right)\cong 0$.

16 $$m\frac{{d}^{2}x(t)}{d{t}^{2}}+\beta \frac{dx\left(t\right)}{dt}={-\upomega }_{0}^{2}\left[1+\varepsilon cos\left({\Omega }_{0}t+\varphi \right)\right]x.$$

Equation (15) refers tothe “low friction range”, i.e., when the system’s friction force is negligible. In such a case, concerning the double frequency parametric resonance condition, the resonance frequency window extends between ${2\omega }_{0}-\frac{1}{2}\epsilon {\omega }_{0}<$ ω $<{2\omega }_{0}+\frac{1}{2}\epsilon {\omega }_{0}$. As above stressed, in the presence of friction the solutions include multiplicative exponential damping factors ${e}^{{\alpha }_{i}t}$ and these terms give rise to a decrease of the resonance windows.

In the following we shall also assume that the constant phase $\varphi$ is zero.

Let us take into account the energy balance between the incoming ${R}_{in}$ and outcoming ${R}_{out}$ radiation.

In particular, it is:

17 $$ R_{in} = Q(1 + A\cos {\Omega }_{0} t) $$

where $Q$ is the solar constant and A is the amplitude of the modulation, estimated, by Milankovitch, to be equal to 5 × 10^−4^, with ${\Omega }_{0}=\frac{2\pi }{T}=\frac{2\pi }{100000 years}$.

Furthermore, it is:

18 $$ R_{out} = \mu \left( T \right)R_{in} + E\left( {T_{E} } \right) $$

where $\mu \left(T\right)$ is the albedo, i.e., the reflected solar radiation which, for simplicity, has a linear decreasing trend with temperature, and $E\left({T}_{E}\right)=4\pi {R}^{2}\sigma {{T}_{E}}^{4}$, with $\sigma$ Stefan-Boltzmann constant $\sigma =5.67*{10}^{-8}W/{m}^{2}/{K}^{4}$, is the energy radiated by the Earth according to its equilibrium temperature which results ${T}_{E}=255K=-18^\circ C$.

As far as albedo is concerned, we can assume a decreasing linear behaviour:

19 $$ \mu \left( T \right) = a - bT $$

Literature data for the straight-line parameters furnish the following values $a=1\div 1.2, b=1$. Then, denoting with c the Earth’s thermal capacity it is:

20 $$ \begin{gathered} c\frac{dT}{{dt}} = R_{in} - R_{out} = \hfill \\ Q(1 + A\cos \Omega_{0} t) - \mu \left( T \right)Q(1 + A\cos \Omega_{0} t) - E\left( {T_{E} } \right) \hfill \\ = Q(1 + A\cos \Omega_{0} t)\left( {1 - \mu \left( T \right)} \right) - E\left( {T_{E} } \right) = \hfill \\ Q\left( {1 + A\cos \Omega_{0} t)\left( {1 - (a - bT} \right)} \right) - E\left( {T_{E} } \right) = \hfill \\ bQ\left( {1 + A\cos \Omega_{0} t} \right)T + \left( {1 - a} \right)AQ\cos \Omega_{0} t + Q - Qa - 4\pi R^{2} \sigma T_{E}^{4} \hfill \\ \end{gathered} $$

And being $a=1\div 1.2, b=1$, it results:

21 $$c\frac{dT}{dt}=Q\left(1+Acos {\Omega }_{0}t\right)T-4\pi {R}^{2}\sigma {{T}_{E}}^{4}$$

Turning back to the complete equation we have:

22 $$c\frac{{d}^{2}T}{d{t}^{2}}+c\frac{dT}{dt}=Q\left(1+Acos {\Omega }_{0}t\right)T-4\pi {R}^{2}\sigma {{T}_{E}}^{4}$$

By calculating the second derivative of eqn, it is:

23 $$c\frac{{d}^{2}T}{d{t}^{2}}=Q\frac{dT}{dt}+AQ\frac{dT}{dt}cos {(\Omega }_{0}t)-{\Omega }_{0}AQTsin\left( {\Omega }_{0}t\right)$$

Under the hypothesis of negligible dissipation, i.e. $c\frac{dT}{dt}=0$, the Eq. (28) becomes:

24 $$c\frac{{d}^{2}T}{d{t}^{2}}=-{\Omega }_{0}AQTsin\left( {\Omega }_{0}t\right)$$

which is the equation of a parametric resonator with negligible damping effects.

The stated equation furnishes an oscillatory solution whose amplitude can vary in time. Under the hypothesis of a slowly varying oscillation amplitude, within specific frequency ranges such an amplitude results an increasing function of time and parametric resonance effects occur. In the presence of friction, within the resonance frequency ranges, multiplicative exponential damping factors are to be included in the solution and this causes the resonance windows to shrink. In other words the range of the permitted resonance conditions are reduced when dissipative effects are present.

## Results and discussion

**Figure 5:**
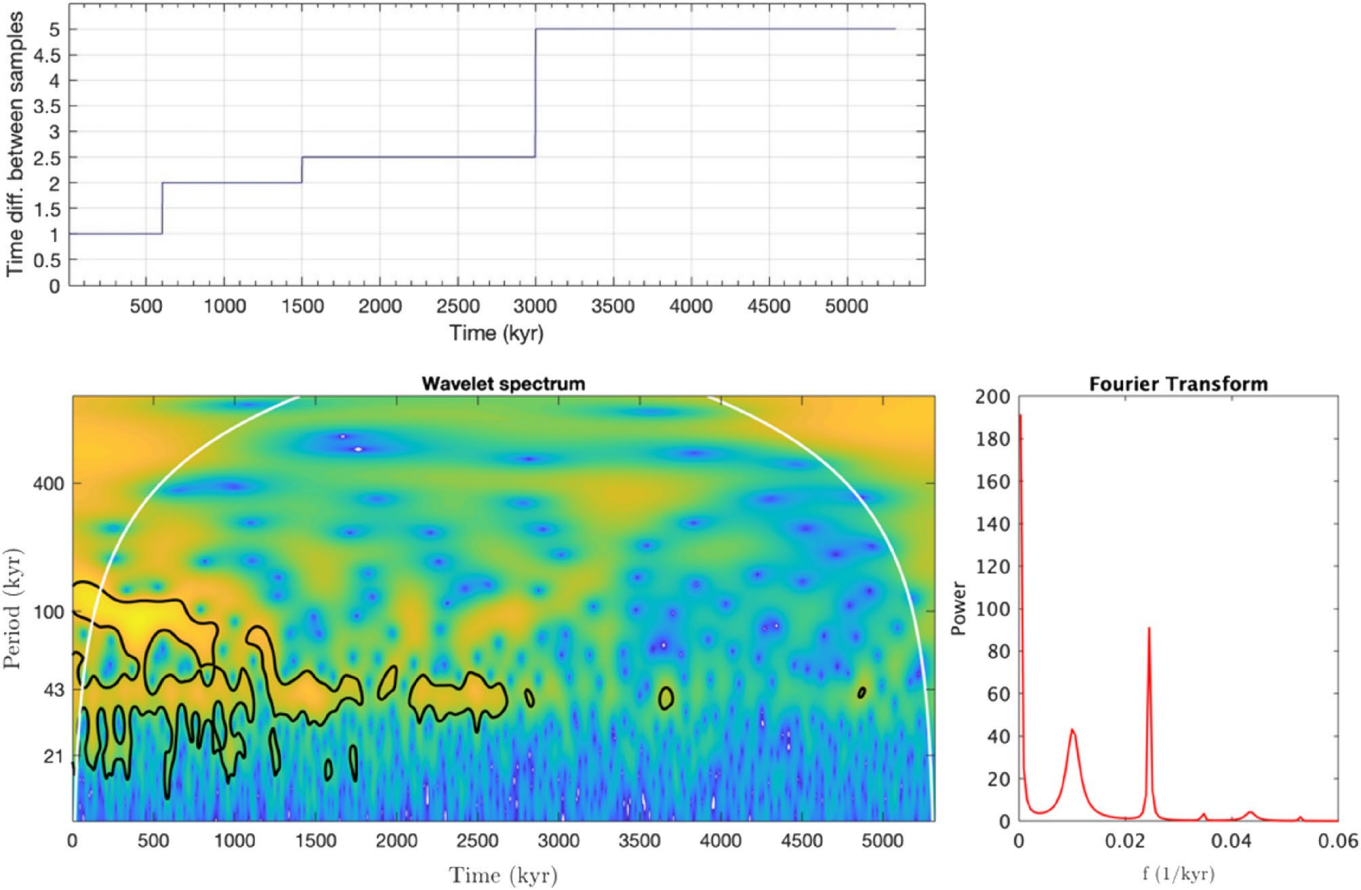
On the top: the peculiar operated choise for the signal sampling; in particular, going back to the past, up to 600 Kyr the sampling step was chosen to be equal to 1 kyr; it becomes 2 kyr from 600 Kyr to 1500 kyr; then it becomes 2.5 Kyr from 1500 to 3000 Kyr; finally, in the region 3000–5000 Kyr, it becomes 5 Kyr. On the right: Fourier power spectrum as a function of frequency, expressed in inverse Kyr, of the whole not equispaced temperature variation record when the Lomb–Scargle approach is employed. On the left: Wavelet transform scalogram of temperature variation data reported in Fig. 1, obtained by using an analytical Morlet wavelet mother function with parameter 6.

For the reconstructed temperature variation record reported in Figs. 1, 5 displays, on the top, the peculiar operated choise for the signal sampling; on the right, the fitting result of the Lomb-Scargle method-calculated Fourier power spectrum of the nonequispaced temperature data set. On the left, the figure shows the wavelet scalogram of the temperature record. With respect to the Fourier spectrum, this latter allows one to localize, in time, the frequency components of the temperature variations over the past 5.5 million years. More specifically, the scalogram of the temperature variation data has been produced by using an analytical Morlet wavelet with parameter 6 and is reported in Fig. 5^50–52^. In particular, going back to the past, up to 600 kyr the sampling step was chosen to be equal to 1 kyr; it becomes 2 Kyr from 600 to 1500 kyr; then it becomes 2.5 kyr from 1500 to 3000 Kyr; finally, in the region 3000–5000 Kyr, it becomes 5 Kyr.

Figure 5 shows that the 100 Kyr cyclicity is significantly reduced before ~ 1.4 Myr, whereas the 41 Kyr periodicity extends longer. Furthermore, the registered cyclicities fluctuate within a wide range.

Both the applied protocols, i.e., The FT and WT analyses confirm the following deductions for the three most important contributions: i) a temperature oscillation contribution occurs with a period of 98.425 Kyr that corresponds to the period of the Earth's orbital eccentricity; ii) a temperature oscillation contribution is also present with a period of 40.8163 Kyr corresponding to the period of the Earth's axis tilt; and iii) a temperature oscillation contribution with a period of 23.020 Kyr that corresponds with the Earth's axis shifting during the solar precession is present. Furthermore, WT analysis allows to clearly reveal the periodicity of about 400 Kyr centered at ~ 3.4 Myr (see Fig. 5), whereas the signal in the extreme regions of the scalogram time window are not significant since they are affected by the WT influence cone^53,54^.

In a future work, data on millennial climate cycles from various sources will be taken into account in order to provide a more comprehensive and universal case.

The physical mechanisms underpinning these main glacial and interglacial cycles remain unclear in many regards. The glacial-interglacial shifts, for example, take place around every 100 Kyr, but the variations in insolation forcing are relatively small in this frequency range. Similarly to this, a very warm interglacial period around 400.000 years ago took place when insolation fluctuations were at their lowest and, in this framework, Paillard showed that multiple states play an important role for the climate system.

More in details, starting from the temperature variation vs time record, we first calculated its FT spectrum, and then, for the obtained FT spectrum, we performed a fit by using six Lorentzian contributions:

25 $$y = {y}_{0}+\sum_{i=1}^{6}\left(2\frac{{A}_{i}}{\pi } \right)\left(\frac{{w}_{i}}{4{\left(f-{f}_{i}\right)}^{2}+{w}_{i}^{2}}\right)$$

where ${{\varvec{A}}}_{{\varvec{i}}}$ is the area of the ith Lorentzian contribution, ${{\varvec{f}}}_{{\varvec{i}}}$ is its center frequency, ${{\varvec{w}}}_{{\varvec{i}}}$ is its width and, finally, ${{\varvec{y}}}_{0}$ is the background value that in the present case is ${{\varvec{y}}}_{0}=0$.

Table 1 shows the obtained fitting parameter values for the six Lorentzian curves. The contribution of the first Lorentzian curve, which is centered at ${{\varvec{f}}}_{{\varvec{i}}}=0$, is not taken into account; in other words, we consider only the five Lorentzian contributions indicated with ${\varvec{i}}=2,3,4,5,6.$

**Table 1:** Fitting parameter values for the six Lorentzian curves.

	1	2	3	4	5	6
${A}_{i}$	9.285	0.205	0.070	0.010	0.015	0.003
${f}_{i}(k{y}^{-1})$	0.00000	0.01016	0.02450	0.03450	0.04344	0.05268
${w}_{i}(k{y}^{-1})$	1.39e−05	0.003	4.84e−04	1.14e−04	0.0022	1.04e−04

Table 2 shows in the first column the values of the frequency centers of the Lorentzian curves ${{\varvec{f}}}_{{\varvec{i}}}$, with *i* = 2, 3, 4, 5, 6, excluding the first Lorentzian centered at frequency ${{\varvec{f}}}_{{\varvec{i}}}=0;$ in the second and third columns the minimum ${{{\varvec{f}}}_{{\varvec{i}}\boldsymbol{ }}}_{{\varvec{m}}{\varvec{i}}{\varvec{n}}}$ and maximum ${{{\varvec{f}}}_{{\varvec{i}}\boldsymbol{ }}}_{{\varvec{m}}{\varvec{a}}{\varvec{x}}}$ values of the frequencies as evaluated from the Lorentzian widths; in the fourth and fifth columns the respective values of the maximum ${{{\varvec{T}}}_{{\varvec{i}}\boldsymbol{ }}}_{{\varvec{m}}{\varvec{a}}{\varvec{x}}}$ and minimum ${{{\varvec{T}}}_{{\varvec{i}}\boldsymbol{ }}}_{{\varvec{m}}{\varvec{i}}{\varvec{n}}}$ values of the periods; in the sixth column the estimated variations of the periods $\Delta {{\varvec{T}}}_{{\varvec{i}}}$; in the seventh, eighth and nineth column the first three parametric resonance frequencies obtained by means of the formula ${{{\varvec{f}}}_{{\varvec{i}}\boldsymbol{ }}}_{{\varvec{n}}}=\frac{2{{\varvec{f}}}_{{\varvec{i}}}}{{\varvec{n}}}\boldsymbol{ }(\mathbf{w}\mathbf{i}\mathbf{t}\mathbf{h}\mathbf{n}=1,2,3),$ and, finally, in the tenth, eleventh and twelfth and columns the respective values of the parametric resonance periods ${{{\varvec{T}}}_{{\varvec{i}}\boldsymbol{ }}}_{1}$, ${{{\varvec{T}}}_{{\varvec{i}}\boldsymbol{ }}}_{2},\boldsymbol{ }{{{\varvec{T}}}_{{\varvec{i}}\boldsymbol{ }}}_{3}$.

**Table 2:** Frequency values, ${{\varvec{f}}}_{{\varvec{i}}},$ extracted from the fitting procedure of the Fourier transform of the Vostok temperature variation record versus time, frequency and period minimum and maximum values ${{{\varvec{f}}}_{{\varvec{i}}\boldsymbol{ }}}_{{\varvec{m}}{\varvec{i}}{\varvec{n}}}$*, *${{{\varvec{f}}}_{{\varvec{i}}\boldsymbol{ }}}_{{\varvec{m}}{\varvec{a}}{\varvec{x}}}$*, *${{{\varvec{T}}}_{{\varvec{i}}\boldsymbol{ }}}_{{\varvec{m}}{\varvec{a}}{\varvec{x}}}$*,* ${{{\varvec{T}}}_{{\varvec{i}}\boldsymbol{ }}}_{{\varvec{m}}{\varvec{i}}{\varvec{n}}}$, period variation $\Delta {{\varvec{T}}}_{{\varvec{i}}}$ together with the first three (with *n* = *1, 2, 3*) parametric resonance frequencies ${{{\varvec{f}}}_{{\varvec{i}}\boldsymbol{ }}}_{{\varvec{n}}}=\frac{2{{\varvec{f}}}_{{\varvec{i}}}}{{\varvec{n}}}\boldsymbol{ }(\mathbf{w}\mathbf{i}\mathbf{t}\mathbf{h}\mathbf{n}=1,2,3)$ and periods ${{{\varvec{T}}}_{{\varvec{i}}\boldsymbol{ }}}_{1}$, ${{{\varvec{T}}}_{{\varvec{i}}\boldsymbol{ }}}_{2},\boldsymbol{ }{{{\varvec{T}}}_{{\varvec{i}}\boldsymbol{ }}}_{3}$.

${f}_{i}({Kyr}^{-1})$	${{f}_{i }}_{min}({Kyr}^{-1})$	${{f}_{i }}_{max}({Kyr}^{-1})$	${{T}_{i }}_{max}(Kyr)$	${{T}_{i }}_{min}(Kyr)$	$\Delta {T}_{i}(Kyr)$	${2f}_{i}({Kyr}^{-1})$	${\frac{2}{2}f}_{i}({Kyr}^{-1})$	${\frac{2}{3}f}_{i}({Kyr}^{-1})$	${{T}_{i }}_{1}(Kyr)$	${{T}_{i }}_{2}(Kyr)$	${{T}_{i }}_{3}(Kyr)$
0.01016	0.007106	0.01316	140.7261	75.9878	64.7383	0.02032	0.01016	0.00677333	49.2125984	98.4250	147.637868
0.02450	0.024016	0.024984	41.6389	40.0256	1.6133	0.04900	0.02450	0.01633333	20.4081633	40.8163	61.2245023
0.03450	0.034386	0.034614	29.0816	28.8900	0.1916	0.06900	0.03450	0.023	14.4927536	28.9855	43.4782609
0.04344	0.04124	0.04564	24.2483	21.9106	2.3377	0.08688	0.04344	0.02896	11.5101289	23.0202	34.5303867
0.05268	0.05257	0.05278	19.0223	18.9466	0.0757	0.10536	0.05268	0.03512	9.49126803	18.9825	28.4738041

It should be taken into account that although the maximum parametric resonance effect occurs when it is n = 1, i.e., ${{{\varvec{f}}}_{{\varvec{i}}\boldsymbol{ }}}_{1}=2{{\varvec{f}}}_{{\varvec{i}}}\boldsymbol{ },$ that is, the most significant parametric oscillation energization occurs when the modulation cycle is repeated twice during a period of natural oscillation, parametric resonance effects are also possible when the parameter changes once during a period, twice during three periods, and so on.

An inspection of the values reported in Table 2 shows that, as a consequence of the large width of the first Lorentzian contribution $\Delta {{\varvec{T}}}_{1}$ that reflects itself into the wide allowed range for ${{\varvec{T}}}_{1}$, the first-order resonances relative to the first cycle, ${{\varvec{\Omega}}}_{0}$, could be coupled with the second one ${{\varvec{\Omega}}}_{1}\approx 2{{\varvec{\Omega}}}_{0}$, giving rise to an enhanced resonance effect that corresponds to the 10^5^ year Milankovitch cycle. On the other hand, the second cycle, ${{\varvec{\Omega}}}_{1}$, and the third cycle, ${{{\varvec{\Omega}}}_{2}\approx 2{\varvec{\Omega}}}_{1}$, could be coupled to each other, giving rise to an enhanced resonance effect that corresponds to the 40.8 10^3^ years Milankovitch cycle.

In synthesis, the three Milankovic’s frequencies are connected by the following relations:

26 $${\Omega }_{0}=\frac{2\pi }{{T}_{1}}\approx \frac{2\pi }{98.4 kyr}{{;\Omega }_{1}\approx 2\Omega }_{0}=\frac{4\pi }{{T}_{1}}\approx \frac{2\pi }{40.8 kyr};{{\Omega }_{2}\approx 2\Omega }_{1}=\frac{8\pi }{{T}_{1}}\approx \frac{2\pi }{23.02 kyr}$$

It should be taken into account that, even if the allowed parametric resonances could be considered weak, due to the width of the Lorentzian contributions whose effects extend beyond the curve half-width at half maximum with respect to the center frequency values and because the energizing effect increases over time with a speed proportional to the energy of the system itself, and therefore exponentially, the amplifying effect can be considerable. In other words, the climate parametric resonance model introduces a set of positive feedback loops in which the system response is amplified with a net positive gain. This determines a self-reinforcing feedback that amplifies the effects of the small disturbances connected with Milankovick’s cycles. These climate forcings push the climate system in the direction of periodic warming and cooling.

In this framework, it should be noticed that the effect of a surface fluid temperature increase during the resonance condition is the exacerbation of extreme weather events; in fact, heat from the planet’s surface and from sub-surface acts as a source of energy making extreme weather events stronger and longer-lasting. Nevertheless, following the adopted model, the shift of the orbital parameter values from the resonance conditions, restores the previous climate phase, so giving rise to the climate cycle.

It should be taken into account that, unlike other climate forcings, the parametric resonance effect furnishes a contribution that is external to the Earth system and internal to the solar system.

For instance, Earth’s internal feedback is furnished by increased atmospheric concentrations of greenhouse gases, which causes warming at the surface; the main positive internal feedback in global warming is the tendency of warming to increase the amount of water vapor in the atmosphere, which in turn leads to further warming. On the other hand, negative Earth’s internal feedback comes out from the Stefan–Boltzmann law, i.e., by the amount of heat radiated from the Earth into space.

The parametric resonance effect acts on the climate system as an internal solar system effect and as an external Earth effect and plays an important role since the energy in the system increases over time with a speed proportional to the energy of the system itself and therefore exponentially. As a result, the parametric resonance condition effect will increase over time.

The significant reduction in temperature of approximately 6 °C ÷ 10 °C that distinguishes the transition from the interglacial to the glacial epoch cannot be accounted only by differences in the solar radiation distribution caused by the 10^5^ year Milankovitch cycle. To take into account positive feedback effects that are able to magnify the impact of this cycle, a parametric resonance model for climate is proposed, according to which the system's energization results from periodic fluctuations of the system's internal parameters. On this concern, it should be noted that temperature changes are primarily associated with variations in incoming summer radiation, which are determined not only by changes in the Earth's eccentricity orbit, but also by variations in the axis's inclination; according to the Stefan-Boltzmann law, the extreme values of summer insolation correspond to a temperature variation of about 3.85 °C, and when the unequal albedo for the extreme conditions is considered, this temperature variation can increase. However, in the present work it is shown that, following the parametric resonance climate model, the system energization, due to periodic variations in the internal solar system parameters, is exponential. In particular, the parametric resonance model works just when weak oscillations with opportune frequencies are present in the system and has the peculiarity that even small energy variations increase over time proportionally to the energy of the system itself, i.e., exponentially at frequency values of approximately ${{\varvec{\Omega}}}_{{\varvec{n}}}=\frac{2{{\varvec{\omega}}}_{0}}{{\varvec{n}}}$ ($\mathbf{n}=1,2,3$…).

It should be noticed that although the solar system resonances are weak, due to the fact that the ratio between amplitude values is the same changing the time variable by the same amount, the amplification is exponential. In other terms, the energizing effect increases over time with a rate proportional to the energy of the system itself and hence in an exponential way.

## Methods

### Fourier transform and wavelet transforms

The Fourier transform (FT) and Wavelet Transforms (WT) are two of the many techniques used to analyse time signals in the frequency space.

FT is well known to be based on specific collections of sine or cosine functions that offer a signal representation in terms of a typical orthogonal basis. The assumptions of linearity and stationarity, as well as the a priori selection of basis functions that do not vary over time, are some of the FT limitations. As a result, the basis projection yields a "global" frequency analysis, which might not be a good match for nonstationary processes. To solve the nonstationarity issue and provide simultaneous time–frequency analysis, some strategies have been proposed. Windowed FT analysis and WT are two of the proposed methods. Windowed FT analysis performs an FT analysis on a sliding window that is shorter in length than the input data set, after which each windowed subset of the data is subjected to a projection.

On the other hand, WT analysis is based on a collection of orthogonal functions that disappear outside of a finite interval and have compact or nearly compact support. The interval is rescaled to highlight various time scales, and the interval center can be moved to take nonstationarity into consideration. The decomposition coefficients are still calculated using a projection-based approach, but the final coefficients rely on the size, shape and placement of the interval of support. Dilation and translation can be used to create a set of orthonormal functions from a "mother" wavelet.

From a formal point of view, FT constitutes an extension of the Fourier series, which furnishes advantages for functions $f\left(t\right)$ that are nonperiodic, i.e., when the period $T\to \infty$. FT decomposes the function $f\left(t\right)$ into sets of sine and/or cosine functions or exponential functions ${e}^{2\pi i\omega t},$ each with weight $\widehat{f}\left(\omega \right),$ with continuously variable frequencies $\omega$:

27 $$f\left(t\right) = {\int }_{-\infty }^{+\infty }\widehat{f}\left(\omega \right){e}^{2\pi i\omega t}dt$$

FT suffers from the limitation that it does not allow a connection between the frequency spectrum and the signal evolution in time. To overcome this difficulty, WT analysis has been formulated^28^. The WT approach allows the decomposition of a signal into its wavelet components by means of the mother wavelet function $\psi$:

28 $${f}_{\psi }\left(a, \tau \right) = \frac{1}{\sqrt{a}}{\int }_{-\infty }^{+\infty }f\left(t\right){\psi }^{*}\left(\frac{t - \tau }{a}\right)dt$$

where $a>0$ denotes the scale, i.e., the inverse of the frequency, $\tau$ represents the shift of the scaled wavelet along the time axis, and ${\psi }^{*}$ represents the complex conjugation of the mother wavelet. Then, the scaled and shifted mother wavelet ${\psi }_{a,\tau }\left(t\right)$ can be expressed by.

${\psi }_{a,\tau }\left(t\right) = \frac{1}{\sqrt{a}}\psi \left(\frac{t - \tau }{a}\right).$ In contrast to FT, which displays only the signal frequencies that are present, WT also displays their time location. In addition, WT takes into account several wavelet mother functions; common characteristics of mother wavelets include orthogonality, compact support, and symmetry. Different mother wavelet functions have distinct effects when applied to the same signal. This makes picking the finest mother wavelet extremely important. A comparison of the various levels of correlation between the signal and several mother wavelet functions was carried out in the present study^29^. In the present work, to analyse the time series constituted by the Earth’s temperature variations, the Morlet wavelet was thus selected as the mother wavelet $\psi \left(t\right) = {e}^{\left(i{\omega }_{0} - \frac{{t}^{2}}{2{\sigma }^{2}}\right)},$ where ${\omega }_{0}$ represents the center pseudofrequency and $\sigma$ provides the wavelet bandwidth. It should be noted that in the special case in which the wavelet mother is $\psi \left(t\right)={e}^{-2\pi it}$, the WT transform reduces to the FT. For the present study, the MATLAB environment was used.

The data registered at the Vostok station support the hypothesis that Milankovitch cycles regulate the variations in glacial and interglacial temperatures and that these variations are also influenced by atmospheric concentrations of greenhouse gases.

As a matter of fact, the variations in the solar radiation distribution during the Milankovitch cycles alone cannot explain the sharp drop in temperature of approximately 10 K that marks the transition from the interglacial to the glacial age. In fact, temperature variation calculations on the basis of the ~ 10^5^ years Milankovitch cycle furnish a temperature variation of only 0.2 K ÷ 0.3 K.

## Data Availability

The data of Fig. 1 are available within the article : Lisiecki, L.E. & Raymo, M.E. A Pliocene-Pleistocene stack of 57 globally distributed benthic δ ^18^O records A Pliocene-Pleistocene stack of 57 globally distributed benthic. Palaeoceanogr Paleoclima 20, PA1003 (2005) 10.1029/2004PA001071 and they can be downloaded from https://lorraine-lisiecki.com/LR04stack.txt.
